# Management of Portal Vein Thrombosis in Cirrhotic Patients

**DOI:** 10.4084/MJHID.2009.014

**Published:** 2009-11-25

**Authors:** Lucio Amitrano, Maria Anna Guardascione

**Affiliations:** UOC di Gastroenterologia, Ospedale A. Cardarelli, Napoli, Italy

## Abstract

Portal vein thrombosis (PVT) not associated with hepatocellular carcinoma is considered a frequent complication of liver cirrhosis but, unlike PVT occurring in non-cirrhotic patients, very few data are available on its natural history and management. The reduced portal blood flow velocity is the main determinant of PVT but, as in other venous thromboses, multiple factors local and systemic, inherited or acquired often can concur with. PVT has a variety of clinical presentations ranging from asymptomatic to life-threatening diseases like gastroesophageal bleeding or acute intestinal ischemia. It is usually diagnosed by Doppler ultrasound but computed tomography and magnetic resonance imaging are useful to study the extent of thrombosis and the involvement of the abdominal organs. The risk of bleeding mainly determined by the presence of gastroesophageal varices and clotting alterations causes concern for the treatment of PVT in cirrhotic patients. To date, anticoagulant therapy seems to be indicated only in patients awaiting liver transplantation. This review focuses on the definition of the subgroups of patients with cirrhosis that might benefit from treatment of PVT and examines the pros and cons of the available treatments in terms of efficacy, monitoring and safety, providing also perspectives for future studies.

## Introduction:

Liver cirrhosis is the most frequent underlying disease in patients with non-neoplastic thrombosis occurring in the portal vein (PVT). In a large autopsy series of patients with PVT, neoplasia was present in 67%, cirrhosis in 28% and in only 5% of patients PVT was considered primitive^1^. In view of the recent progress in the knowledge of pathogenetic mechanisms, prognosis and therapeutic strategy of PVT these three clinical conditions have to be considered separately^2^–^6^.

Hepatocellular carcinoma (HCC) is the most frequent cause of PVT in cirrhosis, being present in up to 44% of cases^7^ and it has always to be searched for when a new diagnosis of PVT is made.

This review deals with PVT occurring in patients with liver cirrhosis without HCC focusing on the necessity and/or the opportunity of management of this specific complication of liver disease.

## Prevalence:

The occurrence of PVT in cirrhotic patients has been increasingly reported in recent years. This stems from the different procedures utilized for diagnosis and the different settings of cirrhotic patients studied. The presence of PVT was 0.6% when evaluated by old angiographic studies^8^, 4.4% by ultrasound^9^ and 10%–12% when more sensitive procedures, i.e. computed tomography (CT) scan and magnetic resonance imaging (MRI) are used^10^–^11^.

Moreover, the prevalence of PVT increases with the age and severity of liver disease reaching 15% in patients awaiting liver transplantation^12^–^15^ and 36% in explanted liver at pathological examination^16^.

Few studies have focused on the incidence of PVT. The de novo thrombosis within one year, in patients with liver cirrhosis, ranges from 7.4% to 16%^10^,^17^.

## Etiopathogenesis:

It is generally thought that liver cirrhosis leads to a progressive bleeding tendency because of the complex alteration of the clotting system induced by liver failure but recent studies have demonstrated that liver cirrhosis, causing a reduced synthesis of either pro-coagulant or anticoagulant proteins is still associated with a balanced equilibrium of the clotting system set at a lower level^18^–^22^. A series of acquired or inherited conditions can easily tilt this equilibrium towards either bleeding or thrombosis^23^–^27^.

Epidemiological studies show a prevalence of venous thrombosis of 0.5% - 1%^28^–^31^ in patients with liver cirrhosis is even greater than in the general population, whereas patients with PVT have an elevated risk of developing venous thrombo-embolism outside the splanchnic system^32^.

As in all venous thrombosis^33^–^34^ the pathogenesis of PVT in non-cirrhotic^35^–^36^ and in cirrhotic patients^37^ is multifactorial, even if not all known risk factors can be studied easily in this setting due to the impaired synthetic and metabolic ability of the liver. Congenital or acquired, local or systemic factors can interact causing reduction of blood flow velocity, endothelial damage and clotting unbalance^38^ (Table 1).

In patients with cirrhosis the derangement of liver architecture leads to increased intrahepatic vascular resistance, development of porto-systemic collateral circulation and splanchnic vasodilatation and ultimately to a stagnation of the portal flow, which is considered the main predisposing factor to thrombosis.

Zocco et al have recently demonstrated that the portal flow velocity, measured by Doppler ultrasound, is inversely correlated with the risk of PVT^17^. Patients with portal flow velocity less than 15cm/sec have an incidence of PVT of 47. 8% compared to 2% of those with a velocity > 15 cm/sec. According to most recent hypotheses blood stagnation causes initiation of venous thrombosis through endothelial activation induced by hypoxia secondary to the hemoglobin desaturation^39^. Nevertheless, only about 15% of patients with advanced liver disease develop PVT, so all other risk factors implicated in the pathogenesis of venous thrombosis have been searched for in these patients.

The age, sex of the patient and etiology of liver disease have not resulted as risk factors for PVT in patients with cirrhosis, even if a lower prevalence of PVT was reported in PSC and PBC candidates for liver transplantation^12^.

Local risk factors may trigger thrombosis via endothelial damage as abdominal inflammatory diseases like diverticulitis, pancreatitis, cholangitis. Abdominal surgical interventions, particularly portal-caval shunts and splenectomy, are associated with PVT^40^–^41^. The prevalence of other surgical interventions was found higher in cirrhotic patients with PVT in one study^42^ but not in others^10^,^43^.

Previous bleeding episodes from gastroesophageal varices^11^ and their endoscopic treatment^42^ resulted as the main endothelial risk factors for PVT and the coexistence of inherited thrombophilia increases this risk^37^. It has been hypothesized that thrombosis develops because of the direct endothelial damage or of the bacteremia induced by the procedures^45^. An increased endotoxemia and clotting activation have been demonstrated in the portal blood that may trigger thrombotic process in these patients^46^.

Local ablative therapies, percutaneous ethanol injection and thermotherapy for HCC may induce thrombosis of portal branches close to the tumor^47^–^49^.

The G20210A mutation of the prothrombin is the main inherited clotting abnormality that has been associated with PVT development in liver cirrhosis with a prevalence ranging from 21.4% to 29% and an *Odds ratio* of 5.9^10^,^43^–^44^. In a recent study high levels of factor VIII have been found associated with the risk of PVT in patients without cirrhosis^50^. The contribution of factor VIII to PVT in patients with liver cirrhosis remains to be established since high levels of factor VIII are present in the advanced liver disease.

The contribution of FVL, and antiphospho-lipid antibodies to the pathogenesis of PVT in cirrhosis remains controversial. MTHFRTT677 mutation in the absence of hyperhomocysteinemia is not considered a risk factor of venous thromboembolism^43^,^51^–^54^. Likewise, the role of inherited natural anticoagulant deficiency (antithrombin, protein C and protein S) in PVT of patients with liver cirrhosis is difficult to establish^55^. In fact, in these patients synthetic dysfunction of the liver leads to a significant reduction of these proteins and an inherited deficiency could be suspected only in the presence of positive familial studies.

In rare cases of PVT associated with recurrent venous thrombosis, or severe presentation with intestinal infarction an underlying myeloproliferative disorder can be suspected^56^.

Is it worthwhile performing a complete thrombophilic work-up in the presence of PVT in a cirrhotic patient?

The identification of one or more thrombophilic risk factors in a single patient is interesting for research purposes and it useful in the clinical setting to plan the length of anticoagulant therapy. In fact in patients with “strong” and persistent risk factors anticoagulation has to be prescribed lifelong. In the subgroups of patients with cirrhosis and PVT in which anticoagulation is indicated (see below) the presence of thrombophilia justifies a protracted therapy.

## Clinical Presentation:

From a patho-physiologic standpoint, PVT aggravates portal hypertension with increasing blood flow through collateral veins, which in turn favours bleeding from the esophageal and gastric veins. Even if thrombosis mostly occurs in the main portal trunk it may extend to its intrahepatic branches, splenic vein and into the superior mesenteric vein, leading to mesenteric ischemia or infarction, often lethal in these patients^10^,^57^. In normal subjects the reduction of portal flow induced by PVT is compensated by the increase of hepatic arterial flow (hepatic arterial buffer response) preventing the hypoxic damage. In advanced liver cirrhosis the occurrence of thrombosis is not adequately counterbalanced by arterial buffering^58^.

Moreover, thrombosis of the smaller branches of hepatic and portal veins, frequently found in autopsy samples of cirrhotic livers, may induce hepatocyte apoptosis, contributing to the further deterioration of liver disease^16^.

Whereas theoretically the occurrence of PVT may worsen either liver function or portal hypertension^59^–^60^ the outcome of the cirrhotic patients with PVT and the actual impact of the thrombosis on the natural history of the cirrhosis have not been investigated to date.

In clinical practice the influence of PVT has been evaluated only in the setting of liver transplantation where, even if PVT is not considered an absolute contraindication, it represents a complex challenge for the hepatic surgeon and is the cause of increased post-transplantation morbidity and mortality^11^–^15^.

**Figure 1.**
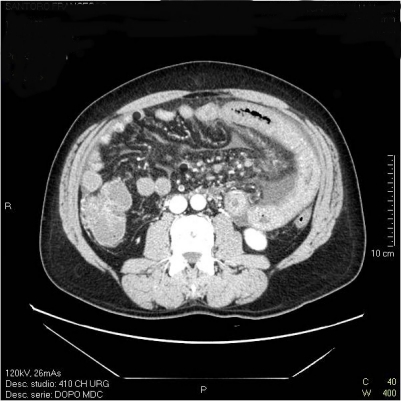
Acute small bowel ischemia due to ptal mesenteric vein thrombosis: see thickened bowel walls, mesenteric oedema and fluid effusion.

PVT can have a variable clinical onset. In a study of 79 cirrhotic patients, PVT caused gastrointestinal bleeding in 39% and abdominal pain in 18%; seventy per cent of patients, admitted for acute abdominal pain, presented intestinal infarction due to the involvement of mesenteric vein (Figure 1).

In 43% of cases PVT was a fortuitous finding during the scheduled ultrasound examination for the screening of hepatocellular carcinoma^10^. In the cirrhotic patients it may be difficult to establish the “age” of thrombosis since the criteria commonly used in non-cirrhotics to define acute or chronic PVT (presence of collateral circulation, presence of signs of portal hyper-tension) are already features of the liver disease.

**Figure 2.**
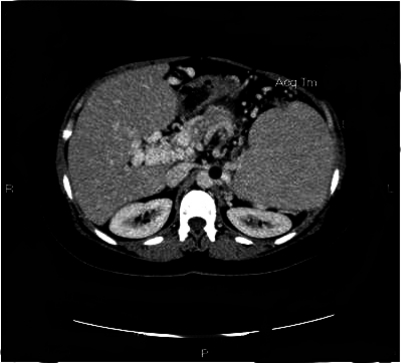
Portal vein cavernoma.

In the previous study^10^ PVT developed in the portal trunk in 56% of cases and concomitant involvement of mesenteric or splenic veins occurred in 29%. Isolate thrombosis of intrahepatic portal branches or of splenic and mesenteric veins were present in less than 15% of cases. At the diagnosis, rarely is the thrombus occlusive and the features of portal cavernoma are less frequent compared to those of non-cirrhotic patients, 21% vs 50% respectively^10^,^61^ (Figure 2).

This is probably due to the earlier diagnosis of PVT and the less pronounced thrombophilic state in patients with cirrhosis.

Nonetheless, the complete occlusion of the mesenteric vein is invariably associated with intestinal ischemia or infarction and the presence of a splanchnic vein thrombosis has to be investigated in cirrhotic patients with acute abdominal pain.

PVT is usually diagnosed by abdominal ultrasound with pulsed and color Doppler examination but this procedure has sensitivity and specificity in detecting the thrombus variable from 66% to 100%, because it is affected both by operator expertise and patient characteristics^62^–^63^. Definitive diagnosis of PVT can be obtained by MRI and CT scan; the first provides a better evaluation of the extent of the thrombosis particularly in the mesenteric vein, reaching a sensitivity and specificity of 98% - 100%^64^. CT scan gives information not only of the extent of the thrombosis and the development of collateral circulation but also of the state of the abdominal organs and it is the procedure of choice when intestinal ischemia or hepatocellular carcinoma are suspected^65^–^66^. In recent studies contrast-enhanced ultrasound^67^ and PET CT proved useful in discriminating between benign or malignant thrombosis^68^.

Overall in the presence of new diagnosis of PVT, the extent of the thrombosis and the clinical features of the patient have to be investigated for a correct evaluation of the prognosis and of the treatment.

## Treatment:

Studies in numerous series of patients with non-cirrhotic PVT have demonstrated that anticoagulation is safe and effective and represents the therapy of choice^61^,^69^–^72^.

Despite the high frequency of PVT in patients with liver cirrhosis, there are very few data on the treatment in this setting^73^–^76^ nor have the recent guidelines on vascular liver disorders addressed this specific issue^6^,^77^.

The main concerns for anticoagulation in cirrhotics arise from the presence of a bleeding risk in these patients and the lack of evidence of a real clinical benefit from the therapy in patients with an advanced liver disease.

In stable conditions the bleeding risk arises mainly from the presence of portal hypertension, in fact more than 50% of these patients have gastroesophageal varices^78^ and anticoagulation therapy might worsen the severity and duration of bleeding episodes.

The utility of the PVT treatment has been assessed only in patients awaiting liver transplantation in whom the presence of a partial PVT represents a cause of increased morbidity and mortality, while a complete thrombosis is a definitive contraindication to transplantation.

In a preliminary study Francoz et al.^11^ demonstrated that anticoagulation therapy achieved a partial or complete portal recanalisation in 42% of 19 patients awaiting liver transplantation. In these patients the survival rate was 79% after OLT, similar to patients without PVT and significantly better than patients with complete portal vein occlusion (50%).

These data, even if necessitating confirmation in larger series of patients, indicated that PVT arising in patients listed for liver transplantation is an indication for anticoagulation therapy.

Since data available are not sufficient to make recommendation on PVT treatment, the following suggestions should be considered advice that can help in clinical practice. The choice of the best management should be evaluated case by case, at least to date.

### Is PVT treatment indicated outside the setting of the patients awaiting liver transplantation?

The presence of acute abdominal pain, due to intestinal ischemia for progression of thrombosis to the mesenteric vein, represents an absolute indication to anticoagulation in the attempt to prevent intestinal infarction. However, there remains to be evaluated whether the presence of mesenteric vein involvement in asymptomatic patients is an indication for treatment. Considering the likelihood of progression of the thrombotic process and the life threatening consequences of the complete mesenteric vein occlusion, treatment is advisable.

It is useful, for clinical purpose, to identify other specific groups of patients in whom PVT treatment needs to be evaluated:

- asymptomatic patients with a compensated liver disease

This group of patients should be treated according to the criteria of non-cirrhotic PVT either to prevent long term complications of the thrombosis or not to preclude a future liver transplantation option. Moreover, a complete assessment of risk factors is suggested in these patients since a thrombophilic state other than cirrhosis per se is likely.

- patients presented with bleeding from portal hypertension

The clinical onset with gastroesophageal bleeding of PVT should not be considered a contraindication to an anticoagulant therapy since the thrombosis may have triggered the bleeding by worsening portal hypertension. Preliminary data show that in these patients anticoagulation therapy is safe if they are adequately treated for bleeding recurrence by medical and endoscopic prophylaxis^79^. The delay in the treatment of PVT till esophageal eradication (median time 4 months) does not hamper the efficacy of the treatment.

In these patients an intrahepatic shunt (TIPS) could be considered as second line treatment because it can achieve the repermeation of the vessel and concomitant treatment of portal hypertensive complications when endoscopic therapies have failed^80^.

Lastly, the treatment of PVT is not to be recommended in patients with advanced liver disease, unsuitable for liver transplantation, since the improvement of survival is unlikely in this setting. Nor it is recommended in the presence of a well established cavernomatous transformation of portal vein in the absence of well defined risk factors.

### Which is the most adequate bleeding prophylaxis in patients with medium-high risk varices candidates to anticoagulation therapy?

Studies addressing the issue in patients with PVTare lacking and it is currently advisable to apply to this group of patients the guidelines for the primary prophylaxis of bleeding followed for patients without PVT^3^ with certain warnings. Medical prophylaxis with non selective beta-blockers is effective in only a portion of patients and has also the disadvantage of further reducing the portal flow^81^. Endoscopic treatment especially sclerotherapy is demonstrated effective as primary prophylaxis of variceal bleeding even if this procedure may be a risk factor for PVT and may induce bleeding complications.

As in non-cirrhotic PVT, anticoagulation therapy is the first line therapeutic strategy in patients with liver cirrhosis. Two main groups of drugs are available to this purpose Vitamin K antagonists (VKA), acenocoumarol and warfarin, and heparins.

Francoz et al.^11^ administered five-day-therapy with low molecular weight heparin (nandroparin 5700 UI/day subcutaneously) followed by acenocoumarol at adjusted doses to achieve an INR value of 2.0 – 3.0 to 19 patients with PVT awaiting liver transplantation. Eight of them (42.1%) obtained recanalisation of portal vein and only one patient had upper gastrointestinal bleeding from post ligation ulceration.

**Figure 3.**
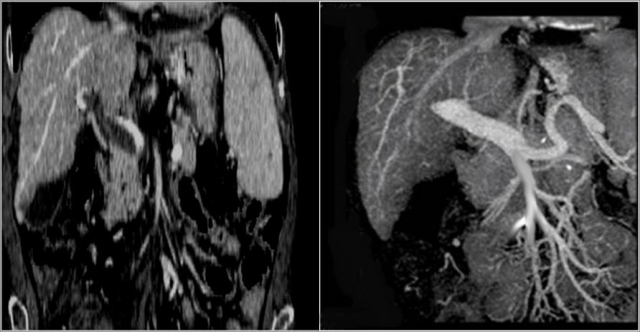
Portal vein thrombosis (left panel); complete recanalisation after anticoagulant therapy (right panel).

Amitrano et al.^79^ treated 28 cirrhotic PVT patients with enoxaparin 200/UI/Kg /day subcutaneously obtaining a complete recanalisation of portal vein after six months in 33.3%, a partial recanalisation in 50% and no response in 16.7% of patients. Further 12 patients who continued anticoagulation obtained complete recanalisation at a median time of 11 months (range 7–17 months), thus a complete response was achieved in 75% of patients. It is worth noting that half of the patients had presented variceal bleeding and had been submitted to variceal endoscopic eradication by band ligation before starting anticoagulation (Figure 3).

The number of platelets did not change significantly in any patient during anticoagulation treatment and only two patients complained of mild anemia due to portal hypertensive gastropathy.

### Which is the best anticoagulant therapy?

In absence of comparative studies, the choice of the anticoagulant therapy has to rely on the following considerations:

-VKAs administered by mouth are more acceptable by the patients but they need periodical dose-adjustments by INR monitoring; in patients with advanced liver disease the INR value is often spontaneously close to or above 2.0 considered a therapeutic range for anticoagulation. Thus INR value as currently measured is inadequate to monitor anticoagulation for patients with severe liver disease^21^,^82^, yet other methods are foreseen^21^,^83^. The long duration of anticoagulant effect due to the long half life of VKAs could represent a further clinical disadvantage in the case of bleeding episode.
- LMWHs have completely replaced unfractionated heparins since they have the same efficacy and do not necessitate monitoring for dose adjustment^84^. Furthermore the fearful heparin induced thrombocytopenia is less common with LMWH and this may be useful in cirrhotic patients with low platelet count.

These disadvantages of both anticoagulation strategies may be overcome by the new oral anticoagulant drugs such as thrombin inhibitors and anti factor Xa^85^.

Currently VKAs may represent a therapeutic option in patients with compensated liver disease who require long term anticoagulation. LMWHs should be given to patients awaiting liver transplantation and to patients with a more severe liver disease.

### Which is the duration of anticoagulation?

The current guidelines for the treatment of venous thromboembolism^86^ can be applied to cirrhotic PVT. After an initial 3–6 month period of anticoagulant therapy patients should be separated into the following groups:

- patients who achieved a complete recanalisation can stop anticoagulant therapy in the absence of previous thrombotic events, or thrombopilic risk factors. They should be followed periodically for PVT recurrence.
- patients with partial recanalisation or with a stable partial thrombosis should continue anticoagulant therapy if candidates for or suitable for future OLT in order to prevent a complete portal vein occlusion.
- patients who failed to achieve any recanalisation of occlusive PVT should stop anticoagulant therapy. Nevertheless, in patients with deep vein thrombosis of the limbs, residual or persistent thrombosis is considered as a risk factor for recurrence requiring long-life anticoagulation^87^–^88^. If this applies to the patients with cirrhotic PVT needs to be evaluated in future follow-up studies.
- patients with recurrence of PVT should be considered for long-life anticoagulant therapy.

Local and systemic thrombolysis has been utilized in the setting of non-cirrhotic venous thrombosis and only rarely in cirrhosis as rescue therapy but high rate of major complications including bleeding has been reported^89^–^90^.

So this therapeutic approach in a cirrhotic patient with PVT should be reserved only in the case of patients with ongoing intestinal infarction not responsive to anticoagulation therapy.

Transjugular intrahepatic porto systemic shunt (TIPS) can be considered a second line treatment of PVT in patients with portal hypertensive complications not controlled by the common therapeutic approach particularly in patients awaiting liver transplantation. In preliminary experience Senzolo et al. reported a successful TIPS placement in a 69% of 13 cirrhotic patients with PVT^80^. This procedure is technically feasible also in patient with cavernomatous transformation of portal vein^91^–^93^ unless intrahepatic portal branches are patent.

## Perspectives:

Portal vein thrombosis is recognized as one of the complications of liver cirrhosis but many aspects still remain to be elucidated. The first is the impact of PVT on the natural history of liver cirrhosis and prospective studies addressing this issue are warranted and to identify subgroups of patients who could benefit from PVT treatment.

The best treatment option in terms of efficacy and safety has also to be established. Furthermore, which anticoagulant treatment, the duration of therapy and the modality of monitoring need to be evaluated in future controlled trials.

## Figures and Tables

**Table 1.** Pathogenetic factors of PVT in cirrhosis

**Risk factors**	**Associated factors**

***Blood flow:*** reduced portal flow velocity	Abdominal surgery
	
***Endothelial :*** abdominal inflammatory diseases, endoscopic variceal treatment, anti-tumoral ablative therapy	Child-Pugh class
	
***Clotting :*** FIIG20210A mutation, elevated levels factor VIII	Previous gastrointestinal bleeding Low platelet count
